# Characterization of a chemostable serine alkaline protease from *Periplaneta americana*

**DOI:** 10.1186/1471-2091-14-32

**Published:** 2013-11-14

**Authors:** Prashant T Sanatan, Purushottam R Lomate, Ashok P Giri, Vandana K Hivrale

**Affiliations:** 1 Department of Biochemistry, Dr. Babasaheb Ambedkar Marathwada University, Aurangabad 431004, MS, India; 2 Plant Molecular Biology Unit, Division of Biochemical Sciences, CSIR-National Chemical Laboratory, Dr. Homi Bhabha Road, Pune 411008, MS, India

**Keywords:** *Periplaneta americana*, Serine alkaline protease, Chemostability, Insect proteases, Industrial catalyst

## Abstract

**Background:**

Proteases are important enzymes involved in numerous essential physiological processes and hold a strong potential for industrial applications. The proteolytic activity of insects’ gut is endowed by many isoforms with diverse properties and specificities. Thus, insect proteases can act as a tool in industrial processes.

**Results:**

In the present study, purification and properties of a serine alkaline protease from *Periplaneta americana* and its potential application as an additive in various bio-formulations are reported. The enzyme was purified near to homogeneity by using acetone precipitation and Sephadex G-100 gel filtration chromatography. Enzyme activity was increased up to 4.2 fold after gel filtration chromatography. The purified enzyme appeared as single protein-band with a molecular mass of ~ 27.8 kDa in SDS-PAGE. The optimum pH and temperature for the proteolytic activity for purified protein were found around pH 8.0 and 60°C respectively. Complete inhibition of the purified enzyme by phenylmethylsulfonyl fluoride confirmed that the protease was of serine-type. The purified enzyme revealed high stability and compatibility towards detergents, oxidizing, reducing, and bleaching agents. In addition, enzyme also showed stability towards organic solvents and commercial detergents.

**Conclusion:**

Several important properties of a serine protease from *P. Americana* were revealed*.* Moreover, insects can serve as excellent and alternative source of industrially important proteases with unique properties, which can be utilized as additives in detergents, stain removers and other bio-formulations. Properties of the *P. americana* protease accounted in the present investigation can be exploited further in various industrial processes. As an industrial prospective, identification of enzymes with varying essential properties from different insect species might be good approach and bioresource.

## Background

Proteases catalyze the hydrolysis of proteins, which leads to the production of small peptides and amino acids [1]. The vast diversity in proteases, regardless of their mode of action and specificity has attracted worldwide attention for using them in biotechnological applications [2]. Recent years have witnessed a significant increase in the use of enzymes as industrial catalysts. The global market for industrial enzymes was estimated around $3.3 billion in 2010. This market is expected to reach $4.4 billion by 2015, with the annual growth rate of 6% over the 5-year forecast period [1]. Proteases represent one of the major groups of enzymes produced and account for 60% of the worldwide sale of the total industrial enzymes [2]. Alkaline proteases hold a big share of the enzyme market and primarily used as detergent additives. Furthermore, alkaline proteases are found to be useful in other industrial sectors, such as leather, food, feed, textile, organic synthesis, pharmaceutical, silk, and for recovery of silver from used X-ray films [3-5]. Although bacterial and fungal [6-8], proteases are used in various industrial applications, insect proteases have comparatively gained lesser attention.

Microorganisms, such as bacteria and fungi remain a major source to obtain industrially important protease till date. In fact, most of the alkaline proteases are especially derived and extensively studied from high yielding strains of *Bacillus species*[5,8]. However, most of the alkaline proteases from microbial species applied for industrial purpose have some limitations, which include low activity and stability towards anionic surfactant (Sodium dodecyl sulphate SDS) and oxidizing agents (bleach and H_2_O_2_) that are common ingredients in modern detergent formulations. And secondly production cost; around 30 to 40% of the production cost of industrial enzymes is accounted for the growth medium of proteases [9]. The industrial demand of highly active preparations of proteolytic enzymes with appropriate specificity and chemostability eventually stimulated the search of alternative protease source.

In this context, insects can act as a promising option for isolating proteases that hold industrially important characteristics. The digestive enzymes of the insects are of interest as a target for insect control and also because of their unusual ability to function in alkaline microenvironment of the gut (pH 10.0 to 12.0) [10]. These observations substantiate that insect gut proteases are finely designed to work optimally in alkaline conditions [11]. Proteolysis is an essential part of food digestion in insects and this process is mediated by the concerted action of several digestive enzymes, which possess the important characteristics such as high temperature and alkaline pH optimum [12,13]. Therefore, these characters of insect proteases are promising while using them for industrial purpose [14]. Previously, we showed that crude gut extract of *P. americana* contains several trypsin and chymotrypsin-like proteases [15]. These enzymes were found to be highly active at alkaline pH therefore could be used as detergent additives. The proteases from *P. americana* and other insect species exhibit highly alkaline optimum pH ranging from 10 to 12. Thus, investigating the potential of *P. americana* gut proteases is of great importance as an industrial point of view. We have selected *P. americana* midgut caecae as a source of study, since it is a factory of diverse proteases, with an objective to exploit properties of these important enzymes.

Here, we report the isolation, purification and characterization of a serine alkaline protease from *P. americana* midgut caecae. The isolated enzyme exhibited stability towards anionic, nonionic surfactants, oxidizing agents and organic solvents. Furthermore, the potential applications of the purified protease in various industries have been discussed.

## Methods

### Material

The following chemicals were obtained from Sigma-Aldrich, St. Louis, MO, (USA): Casein, Sephadex G-100, bovine serum albumin (BSA), phenylmethylsulfonyl fluoride (PMSF), ethylene diamine tetra acetate (EDTA), 5, 5-Dithiobis (2-nitrobenzoic acid) (DTNB) and β-mercaptoethanol. Heavy metal ions, surfactants, organic solvents and chemicals for electrophoresis were purchased from Merck, Germany. Gel X-ray films and medium range molecular mass markers (14.3 to 97.4 kDa) were obtained from Selvas photographic Ltd. Silvassa, India and Genei, Bangalore, India respectively. Commercial detergent powders such as Ariel, Tide, Surf, Ghadi, Henko and Rin were purchased from local market. All other chemicals used were of high analytical grade. *Periplaneta americana* insects were collected from grain godowns in Aurangabad, Maharashtra state India.

### Preparation of enzyme extract

Insects were immobilized by keeping at −20°C for 2 h and dissected mid-ventrally. Midgut caecae were removed and homogenized with pre-chilled mortar and pestle in 1:6 (w/v) volumes of ice-cold 0.1 M Tris–HCl buffer pH 8.0. The homogenate was centrifuged at 10,000 rpm for 20 min at 4°C. The supernatant was collected and divided into 2 mL aliquots and stored at −20°C until use. Protein concentration in the supernatant was measured by Lowry’s method using bovine serum albumin as standard [16].

### Total proteolytic activity assay

Total proteolytic activity was determined by caseinolytic assay [17,18]. Different protein concentrations from *P. americana* caecae extract were added into 2 mL of 0.5% casein (prepared in 0.1 M Tris–HCl buffer pH 8.0) and the mixture was incubated for 20 min at 40°C. After 20 min the reaction was terminated by adding 3 mL of 5% trichloro acetic acid (TCA). The absorbance of TCA soluble peptides was measured at 280 nm.

### Purification steps

#### Acetone precipitation

To the supernatant (50 mL) equal amount of chilled acetone was added and kept overnight at −20°C. Next day the precipitate was collected and centrifuged at 10,000 rpm for 20 min at 4°C. Acetone was removed by air drying and the remaining precipitate was collected. The precipitate was dissolved in minimum amount of 0.1 M Tri-HCl buffer pH 8.0 and centrifuged at 10,000 rpm for 20 min and the supernatant was collected.

#### Gel filtration chromatography

Sephadex G-100 (5 gm) gel was added in 0.1 M Tris–HCl (pH 8.0) and allowed to swell for overnight and column (1.5 × 65 cm) was packed. The column (1.5 × 65 cm) was equilibrated with 0.1 M Tris–HCl buffer pH 8.0. Total 5 mL of acetone precipitate was loaded onto a Sephadex G-100 column (1.5 × 65 cm). The column was eluted with Tris–HCl buffer (pH 8.0) and fractions of 1 mL were collected at a flow rate of 1 mL/min. Protein concentration from each fraction was determined by Lowry method using bovine serum albumin as standard [16].

### Molecular mass determination

Molecular mass of the purified enzyme was determined on 12% sodium dodecyl sulphate polyacrylamide gel electrophoresis (SDS-PAGE) according to the method of Laemmli [19]. Approximately 10 μg of purified protein was loaded on 12% SDS-PAGE with standard molecular mass markers and electrophoresis was carried out at a constant current of 30 mA. After electrophoresis the gel was stained with Coomassie Brilliant Blue R-250 (CBB R-250) and destained to visualize protein bands.

### Visualization of protease isoforms by gel X-ray film contact print method

To visualize the activity of proteases in each purification step, the fractions were separated on 10% native-PAGE and the electrophoresis was carried out at constant current of 20 mA. After PAGE, the gel was equilibrated in 0.1 M Tris–HCl buffer pH 8 for 10 min followed by placing the gel on fresh X-ray film and incubating at 37°C for 30 min [15,20]. The gel was removed and X-ray film was washed with warm water. Protease isoforms in the gel were detected in terms of gelatin hydrolysis of X-ray film, which served as substrate.

### Optimum pH and pH stability of purified protease

The activity of purified enzyme in various buffers was evaluated by adding 20 μL purified enzyme in pre-incubated buffers (1 mL, 0.1 M) of pH ranging from pH 3.0 to 12.0 (0.1 M, pH 3.0 to 12.0 buffers; (i) glycine–HCl pH 3.0, (ii) acetate buffer pH 4.0, 5.0, (iii) phosphate buffer pH 6.0, 7.0, (iv) Tris–HCl pH 8.0, 9.0 and, (v) glycine–NaOH pH 10.0, 11.0, and 12.0) and 1 mL of 0.5% casein dissolved in distilled water. The reaction was carried out at 37°C and terminated after 20 min by adding 3 mL of 5% TCA. The absorbance of TCA soluble peptides was measured at 280 nm. For the determination of pH stability, the purified enzyme was incubated in buffers having different pH ranging from pH 7.0 to 12.0 for 1 h at 40°C and the enzyme activity was determined under standard assay conditions.

### Effect of temperature on activity and stability of the protease

The activity of purified protease at different temperatures (10–100°C) was evaluated by adding 20 μL purified enzyme in 2 mL of 0.5% casein. Reaction mixture and substrate solution were pre-incubated for 30 min at given temperatures before activity measurement. The assay was carried out as mentioned above at different preset temperatures for 20 min. The temperature stability of the purified enzyme was also checked. To examine the temperature stability, the purified enzyme was incubated at different temperatures ranging from 50 to 70°C for 1 h and the residual enzyme activity was measured under standard assay conditions.

### Effects of inhibitors and metal ions on protease activity

The effect of inhibitors on purified protease activity was studied using PMSF (1 mM), EDTA (5 mM), DTNB (5 mM) and β-mercaptoethanol (5 mM) [21]. The reaction mixture was prepared by pre-incubating the purified enzyme with inhibitors for 10 min at 40°C. The protease assay was performed for 30 min at 40°C by above-mentioned method. Protease activity obtained without inhibitor was considered as 100%. Effect of monovalent (Na^+^ and K^+^) and divalent (Cd^2+^ , Zn^2+^, Cu^2+^ , Ba^2+^ and Hg^2+^) metal ions on enzyme activity at a concentration of 5, 10 and 15 mM was investigated by using casein as substrate [21]. The reaction mixture was prepared by pre-incubating the purified enzyme with metal ions at each concentration for 10 min at 40°C and the proteolytic activity was determined for 1 h by above-mentioned method. Enzyme activity without metal ions was considered as 100%.

### Effect of surfactants and oxidizing agents

The suitability of the purified protease as a detergent additive was determined by testing its stability towards surfactants (Tween 20, Tween-80, Triton X-100 and SDS) and oxidizing agents (H_2_O_2_ and bleach). The reaction mixture was prepared by pre-incubating the purified enzyme with surfactant and oxidizing agents at each concentration for 10 min at 40°C and protease activity assay was carried out for 1 h as mentioned above. Enzyme activity without any surfactant and oxidizing agent was considered as 100%.

### Effect of organic solvents

The effect of various organic solvents such as acetone, xylene, benzene, propanol, DMSO, toluene, ethanol, butanol and hexane on purified protease activity was tested. The reaction mixture was prepared by pre-incubating the purified enzyme with each solvent for 10 min at 40°C. Reaction mixture was prepared in 0.1 M Tris–HCl buffer (pH 8.0) to get final concentration of each solvent as 25% and enzyme activity was determined as mentioned for surfactants and oxidizing agents. Enzyme activity without any organic solvent was considered as 100%.

### Stability of protease toward commercial detergents

The stability of purified protease was checked against several commercial detergent powders such as Ariel, Tide, Surf, Wheel, Henko, and Rin. Detergent solutions were prepared in 0.1 M Tris–HCl buffer (pH 8.0) to get final concentration of 1, 10 and 100 mg/mL of the each detergent. Prior to assay detergent solutions were heated at 100°C for 1 h to deactivate the endogenous proteases present in the commercial detergents. The reaction mixture was prepared by pre-incubating the purified enzyme with each detergent with different concentrations for 10 min at 40°C and protease activity was determined by above-mentioned method. The activity of enzyme without detergent was considered as 100%.

### Statistical analysis

All the experiments excluding purification steps were carried out at least three times. Standard deviation and standard error were calculated using MS-Excel.

## Results

### Purification and molecular mass determination

**Figure 1:**
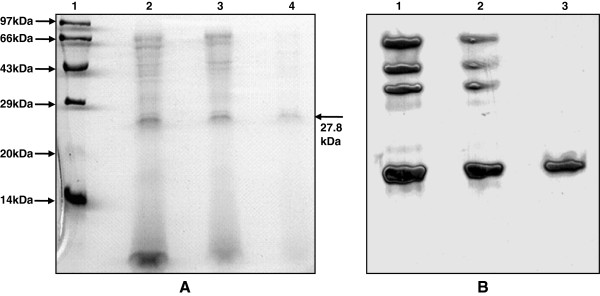
**Purification and molecular mass determination of *P. americana *protease (A).** The purified enzyme was separated on 12% SDS-PAGE and protein bands were stained with CBBR-250. Lane 1, standard molecular weight markers, Lane 2, crude extract, Lane 3, acetone precipitate, Lane 4, gel filtration fraction. **(B)** Detection of protease activity of purified enzyme by gel X-ray film contact print method. Fractions from each purification step were resolved on 10% native-PAGE and protease activity isoforms were visualized on X-ray film. Lane 1, crude extract, Lane 2, acetone precipitate, Lane 3, gel filtration fraction.

The protease from *P. americana* midgut caecae was purified by using acetone precipitation and gel filtration chromatography. The enzyme was purified up to 4.2 fold with 19% recovery. The specific activity of the enzyme was increased up to 4.2 fold after gel filtration chromatography. The results of the purification procedure are summarized in Table 1. The purified fraction was loaded on 12% SDS-PAGE and electrophoresis was carried out. The purified enzyme was appeared as single band on 12% SDS-PAGE when stained with CBBR-250. The molecular mass of the purified enzyme was estimated to be ~27.8 kDa (Figure 1A). The molecular mass of the purified protein was confirmed by the Sephadex G-100 column. The purity of the enzyme was further checked by detecting the protease activity band on X-ray film (Figure 1B).

**Table 1:** **Purification of *P. americana *** **protease**

**Purification**	**Volume (mL)**	**Activity (μM/mL/min)**	**Total activity (μM)**	**Specific activity (μM/mg/min)**	**Total protein (mg)**	**Purification fold**	**Purification yield %**
**Step**							
Crude	50	4.117	164.68	1.52	250	1	100
Acetone ppt	6	1.116	6.669	2.2	30	1.4	4
Gel filtration	5	6.5	32.5	6.5	25	4.2	19

Purification fold, percentage yield and activity of each purification step.

### Optimum pH and stability of the purified protease

**Figure 2:**
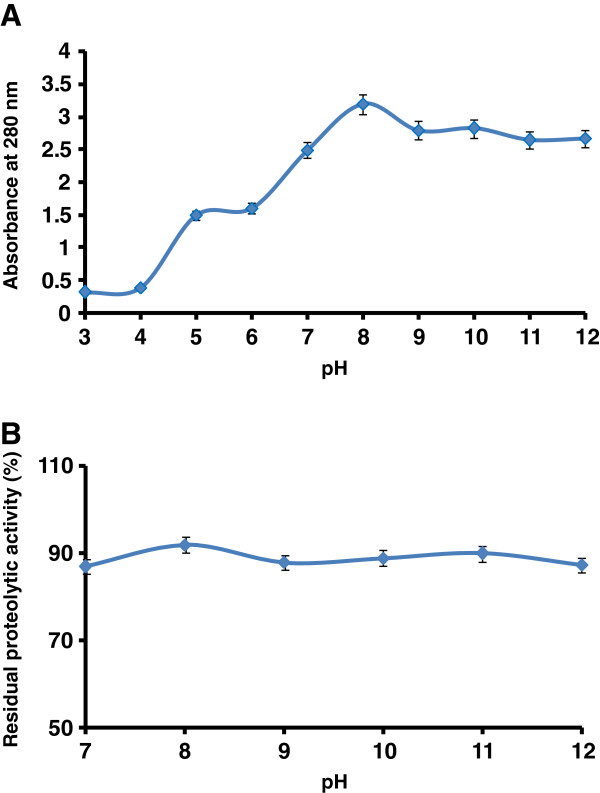
**Effect of pH on (A) activity and (B) stability of the purified *P. americana *protease.** The protease activity was evaluated using different buffers (from pH 3 to 12), whereas the pH stability of the enzyme was determined by incubating the enzyme in alkaline buffers for longer time (1 h). Purified protease showed a significant activity at high alkaline pH range. The error bars show the standard deviation of at least tree replicates.

The effect of pH on purified protease activity was determined over a pH range of 3 to 12. The enzyme was found to be highly active in the pH ranges from 7 to 12, with an optimum pH 8 (Figure 2A). The pH stability profile of the enzyme is shown in Figure 2B. The purified enzyme was observed to be stable at a pH range between 7 and 12 and recovered 90% of its original activity up to 1 h incubation at 40°C.

### Temperature optima and stability

**Figure 3:**
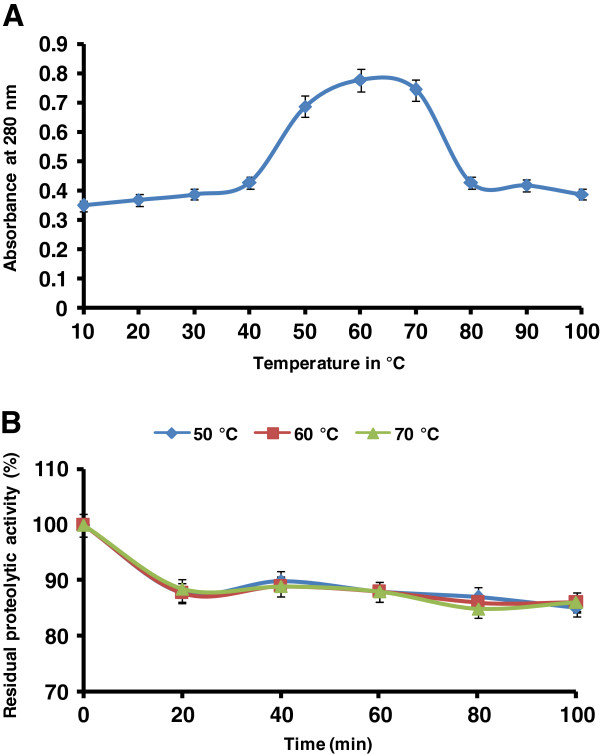
**Effect of temperature on (A) activity and (B) stability of the purified *P. americana *protease.** The temperature optimum was determined by assaying protease activity at different temperature ranges between 10 to 100°C, while the temperature stability was checked at temperature ranges between 50 to 70°C for 1 h. The *P. armigera* purified protease was found to be stable at higher temperature. The error bars show the standard deviation of at least tree replicates.

Effect of temperature on purified protease activity was determined using caseinolytic assay. The enzyme was found active at temperature ranging from 50 to 70°C with an optimum activity at 60°C (Figure 3A). After 70°C the enzyme activity was decreased rapidly. The temperature stability profile revealed that the enzyme was active up to100 min at the temperature between 50 to 70°C. The protease activities relative to control at 50, 60, and 70°C were about 80, 87 and 82% respectively (Figure 3B).

### Effect of inhibitors and metal ions

**Figure 4:**
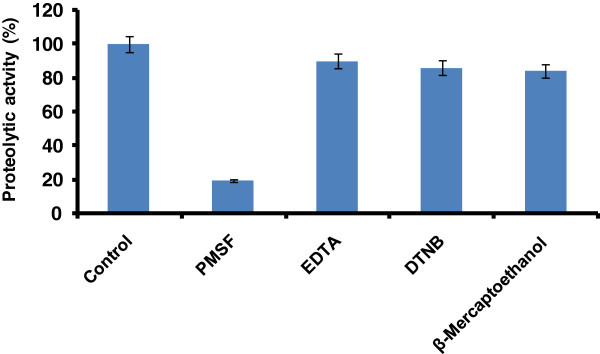
**Effect of inhibitors on the activity of purified *P. americana *protease.** Different inhibitors were pre-incubated with the purified enzyme for 30 min at 40°C and assay was carried out using casein as substrate. The activity of enzyme without inhibitor was considered as 100%. The error bars show the standard deviation of at least tree replicates.

In order to determine the nature of the purified protease, the effect of various inhibitors (PMSF, EDTA, DTNB and β-mercaptoethanol) on enzyme activity was investigated. The enzyme activity was completely inhibited by the serine protease inhibitor PMSF, which indicated that the purified enzyme belongs to serine-type protease family (Figure 4). The thiol reagent DTNB had no influence on the protease activity, while the enzyme activity was slightly inhibited by the chelating agent EDTA and β-mercaptoethanol. The effect of various metal ions (at three different concentrations; 1, 5 and 10 mM) on the activity of purified protease was investigated. Results are summarized in Table 2. Enzyme retained 90% of its activity in presence of Ca^2+^, Zn^2+^, Ba^2+^, Hg^2+^, Cu^2+^, Na^+^, and K^+^ at the concentration 5 mM of each metal ion. However, more inhibition in enzyme activity was recorded with each above-mentioned metal ion at the concentration of 10 mM while about 20% enzyme activity was inhibited at 15 mM concentration.

**Table 2:** **Effect of metal ions on the activity of purified *P. americana *** **protease**

**Metal ion**	**Relative activity (%)**		
	**5 mM**	**10 mM**	**15 mM**
Control	100	100	100
Ca^2+^	95	94	80
Zn^2+^	95	84	80
Cu^2+^	95	75	80
Ba^2+^	94	93	82
Hg^2+^	97	90	80
Na^2+^	92	82	83
K^+^	92	84	85

### Effect of surfactants and oxidizing agents

The effect of anionic surfactant (SDS), nonionic surfactants (Tween-20, Tween-80, Triton X-100) and oxidizing agents (H_2_O_2,_ and bleach) on purified *P. americana* protease activity was investigated and the results are summarized in Table 3. Three different concentrations (1, 5 and 10%) of all above-mentioned reagents were tested against purified protease activity. Enzyme showed 93, 63, and 95% activity in presence of 1% Tween-20, Tween-80 and Triton X-100, respectively, while 60% enzyme activity was found to be retained in presence of these three nonionic surfactants at the concentration of 5 and 10%. Similarly, enzyme was able to retain 60% activity in the presence of 10% SDS. As shown in Table 3, nearly 14% enzyme activity was inhibited by H_2_O_2_ at the concentration of 1%. However, at 5 and 10% concentration H_2_O_2_ inhibited around 37 and 36% protease activity, respectively. Enzyme retained 80% of its activity in presence of 1% bleach, whereas 34 and 40% enzyme activity was inhibited by bleach at 5 and 10% concentration, respectively.

**Table 3:** **Stability of purified *P. americana *** **protease in the presence of various surfactants and oxidizing agents**

**Surfactant/oxidizing agent**	**Relative activity (%)**		
	**1%**	**5%**	**10%**
Control	100	100	100
Tween-20	93	67	61
Tween-80	83	69	64
Triton X-100	95	81	77
SDS	76	64	60
H_2_O_2_	86	63	64
Bleach	80	66	60

### Effect of organic solvents on protease activity

**Figure 5:**
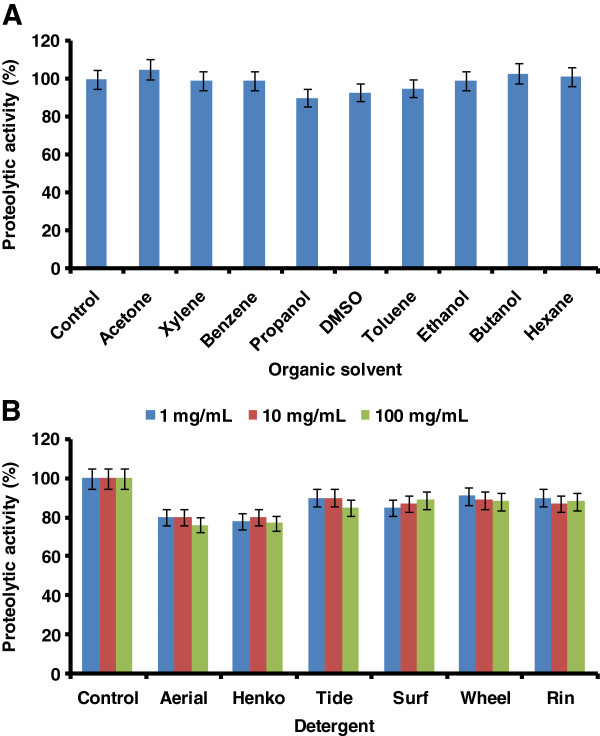
**Effects of various organic solvents on enzyme activity (A).** The effect of organic solvent was determined by incubating the purified *P. americana* protease with different organic solvent for 1 h and the enzyme activity was measured under standard assay conditions. The activity of the enzyme without organic solvent was taken as 100%. The error bars show the standard deviation of at least tree replicates. **(B)** Stability of the purified protease from *P. americana* in the presence of various commercial detergents. The stability with commercial detergents was determined by incubating the purified enzyme with each detergent at different concentrations such as 1, 10 and 100 mg/mL for 1 h at 40°C. Enzyme activity of control sample without any detergent was considered as 100%. The error bars shows the standard deviation of at least tree replicates.

The influence of various organic solvents on the activity of purified protease was studied and obtained result showed that the protease activity was increased up to 5 to 10% in presence of acetone, butanol, and hexane. Around 15% protease activity was inhibited by propanol, DMSO and toluene while enzyme retained its 100% activity in presence of xylene, benzene and ethanol (Figure 5A).

### Stability of protease toward commercial detergents

Effect of various commercial detergents on protease activity was studied at three different concentrations *viz.* 1, 10 and 100 mg/mL. The enzyme retained its 90% activity in presence of Tide, Wheel, Surf and Rin, whereas enzyme showed 80% activity in presence of Ariel and Henko. Similar results were observed at each tested concentration of detergents (Figure 5B).

## Discussion

Proteases with strong activity and stability in highly alkaline pH range are essentially important for biotechnological applications. For example, proteases constitute one of the most important groups of enzymes, which are used as key ingredients in detergent formulation [22]. With the advent of new frontiers in biotechnology, the spectrum of protease application has expanded into many new fields such as clinical, pharmaceutical and analytical chemistry [23-27]. Generally alkaline proteases used in industrial processes are obtained from microorganism; however, these sources have limitations while using in industries. Thus, it is essential to search alternative and reliable sources of proteases with high activity and stability in various conditions. Insect midgut can be an important source to obtain proteases with necessary properties. Previously, we did find high protease activity in the gut of *P. americana*, therefore the present study is an attempt to isolate and characterize chemostable proteases from this important insect species [15].

The alkaline protease from *P. americana* midgut caecae was purified and characterized for its biochemical properties [28-30]. Earlier, Lopez and terra [28] purified a 29 kDa digestive trypsin from *P. americana* and the purification of enzyme was achieved using a combination of two anionic chromatographic steps [28]. Similar protocol was used for the purification of the trypsin from *Tenebrio molitor*[31]. Both above-mentioned studies concluded that, although similar in many instances, *P. americana* and *T. molitor* trypsins differ mainly in their charges. Surprisingly, *P. americana* trypsin binds with anionic resins both at pH 5.0 and 10.0, despite its *pI* of 6.0. Perhaps local surface charges are strong enough to make possible the binding even when the molecule overall charge would be contrary to that. From the observed molecular weight enzyme isolated by us, seems similar to those trypsins isolated from *P. americana* and *T. molitor*. Moreover, *P. americana* protease isolated in the present study, perhaps the known trypsin reported by Lopez and terra [28].

The purified protease was found to be active at alkaline pH (>8). The enzyme also showed good stability in broad pH range i.e. from pH 7 to 12. It has been well known that a large number of proteases present in the insect gut act in high alkaline pH range. Our findings are in accordance with the properties of proteases reported from previous studies; cockroach [15,28,32], *Spilosoma obliqua* (pH 11.0) [33], *Spodoptera litura* (pH 9.0, 10.5, and 11.0) [34], *Heliothis zea* (pH 11.0) [35], *Galleria mellonella* (pH 10.5 and 11.2) [36]*Helicoverpa armigera* (pH 9.5 and 10.0) [37], and *Tenebrio molitor* (pH 8.5) [38]. Most of the insects have midgut pH in the range of 6 to 10. Gut pH conditions are likely to have a major influence on the efficiency of nutrient extraction in insects. The high pH of many insect guts has been attributed to an adaptation of their leaf-eating ancestors for extracting hemicelluloses from plant cell walls [38]. Furthermore, there are some correlations between the midgut enzymes and surrounding symbiotic microflora. Studies from analysis of genomes have concluded that insects do not possess the entire metabolic repertoire to efficiently extract the maximum of nutrients from their food and they depend on their gut microbial community for this purpose [39]. Gut bacteria are unique in the sense that they can thrive in the hostile environment of gut, withstanding extremes of pH and ionic composition and steep redox gradations. Moreover, gut microorganisms are critical to the nutrition, physiology, detoxification and resistance mechanism [40]. Overall, it seems that gut microorganisms have significant contribution in multiple isoforms, diversity and stability of enzymes found in insect gut.

Optimum temperature for activity of alkaline protease was determined to evaluate its suitability for biotechnological applications. The purified alkaline protease exhibited a temperature optimum at 60°C and stability in the temperature range 50 to 70°C. Previously, we reported the temperature optima for proteases in the crude caecae extract of *P. americana* was around 50 to 70°C [15]. The alkaline proteases from other insects also found to be thermostable and their temperature optima were between 50-60°C [34]. For application in detergents and tanning processes, alkaline protease(s) with high temperature and alkaline pH optima are desirable [1,9]. Thus, insect proteases can be suitable candidates for these purposes, as they exhibit required characteristics. Studies on enzyme inhibitors further insights into biochemical properties of the *P. americana* purified protease. The purified enzyme was characterized as a serine protease because its activity was completely inhibited by a specific serine protease inhibitor PMSF. PMSF binds specifically to a serine residue in the active site of serine protease. This is a result of the hyperactivity of serine residue caused by the specific environmental conditions in the enzyme’s active site. Because PMSF binds covalently to the enzyme, the complex can be viewed by X-ray crystallography; it can therefore be used as a chemical label to identify an essential active site serine in an enzyme [41,42]. The chelating agents DTNB, EDTA and β-mercaptoethanol had no influence on the activity of purified enzyme. The high activity of *P. americana* protease in presence of chelating agent is very useful for employing as detergent additive since most of the detergents contain chelating agents as major component.

The purified protease from *P. americana* showed considerable activity at high concentration of various metal ions. Proteases having stability towards higher concentration of metal ions are usually suitable in leather processes, sewage treatment etc. In addition to the stability towards temperature, pH, and metal ions, enzyme used in detergent formulation must be active in presence of surfactants, oxidizing agents and other detergent additive [4,43]. Alkaline proteases from high yielding bacterial strains have been studied extensively. However, few reports are available on the stability of the alkaline proteases towards surfactants and oxidizing agents [44]. Most commonly used proteases such as subtilisin, esperase and savinase are stable in various detergent components but unstable in oxidizing agents [44]. *P. americana* protease exhibited excellent stability and activity against surfactants and oxidizing agents. There is great industrial demand for organic solvent stable proteases to employ in the synthesis of useful pharmaceutical products [45]. Therefore, proteases, which are naturally stable in organic solvents, are essential for synthetic reactions and peptide synthesis and accordingly the protease from *P. americana* exhibited such features.

## Conclusion

Present study deals with the characterization and potential applications of a serine alkaline protease purified from *P. americana*. SDS-PAGE and native gel followed zymographic analyses revealed monomeric nature of the enzyme with a molecular weight of 27.8 kDa. The purified protease was found active and stable in higher temperature and alkaline pH range. Enzyme exhibited excellent stability and compatibility towards detergents, oxidizing and bleaching agents. Obtained results point out that the essential properties of *P. americana* protease can be exploited in various important bioformulations and industrial applications. Altogether, insects appear to be a potential source to obtain industrially important proteases. Protein engineering has played a central role in improving commercially important enzymes and in finding new applications of proteins quite different from their natural function. In future, protein engineering will offer possibilities of generating proteases with entirely new functions. Hence, although alkaline proteases already play an important role in several industries, their potential is much greater and their applications in future processes are likely to increase in the near future.

## Competing interests

The authors declare that they have no competing interests.

## Authors’ contributions

PTS, PRL and VKH designed the research and wrote the manuscript. PTS and PRL performed the research. APG helped in data analysis and writing of the manuscript. All authors read and approved the final manuscript.
